# Excess caffeine exposure impairs eye development during chick embryogenesis

**DOI:** 10.1111/jcmm.12260

**Published:** 2014-03-17

**Authors:** Zheng-lai Ma, Guang Wang, Xin Cheng, Manli Chuai, Hiroshi Kurihara, Kenneth Ka Ho Lee, Xuesong Yang

**Affiliations:** a Department of Histology & Embryology, Key Laboratory for Regenerative Medicine of the Ministry of Education, Institute of Fetal-Preterm Labor Medicine, Medical College of Jinan UniversityGuangzhou, China; b Division of Cell and Developmental Biology, University of DundeeDundee, UK; c Institute of Traditional Chinese Medicine and Natural Products, Jinan UniversityGuangzhou, China; d Key Laboratory for Regenerative Medicine of the Ministry of Education, School of Biomedical Sciences, Chinese University of Hong KongShatin, Hong Kong

**Keywords:** caffeine, ROS, chick embryo, retina, Pax6

## Abstract

Caffeine has been an integral component of our diet and medicines for centuries. It is now known that over consumption of caffeine has detrimental effects on our health, and also disrupts normal foetal development in pregnant mothers. In this study, we investigated the potential teratogenic effect of caffeine over-exposure on eye development in the early chick embryo. Firstly, we demonstrated that caffeine exposure caused chick embryos to develop asymmetrical microphthalmia and induced the orbital bone to develop abnormally. Secondly, caffeine exposure perturbed Pax6 expression in the retina of the developing eye. In addition, it perturbed the migration of HNK-1^+^ cranial neural crest cells. Pax6 is an important gene that regulates eye development, so altering the expression of this gene might be the cause for the abnormal eye development. Thirdly, we found that reactive oxygen species (ROS) production was significantly increased in eye tissues following caffeine treatment, and that the addition of anti-oxidant vitamin C could rescue the eyes from developing abnormally in the presence of caffeine. This suggests that excess ROS induced by caffeine is one of the mechanisms involved in the teratogenic alterations observed in the eye during embryogenesis. In sum, our experiments in the chick embryo demonstrated that caffeine is a potential teratogen. It causes asymmetrical microphthalmia to develop by increasing ROS production and perturbs Pax6 expression.

## Introduction

Caffeine is a white crystalline xanthine alkaloid. It was initially isolated in the eighteenth century and recognized to be a stimulant of the central nervous system because of its ability to enhance alertness. The other effects of caffeine include dieresis, increased heart rate and/or blood pressure after consumption [1]. Caffeine is distributed and consumed worldwide in our daily food stuffs, drinks and medicines [2–4]. It is estimated that 70–95% of pregnant women in the West drink at least two cups of coffee everyday [5,6]. However, we still know very little on the possibility that caffeine in excess might induce abnormal foetal development in pregnant women. Many animal experiments have been conducted on the effects of caffeine during pregnancy, but the exact consequence of caffeine on foetal eye development remains uncertain. These issues are of interest not only to academics but also to the general public concerned with their diet and health.

The vertebrate eye is derived from two kinds of ectoderm cells in the embryo—with (1) the lens and cornea develop from surface ectoderm and (2) the retina, iris and ciliary body develop from the anterior neural plate [7]. Neural crest cells are indispensable for eye development: in absence of the contribution of neural crest, ectopic lenses develop far from the retina [8,9]. In addition, the formation and invagination of the optic stalk coincides with the migration of cranial neural crest (CNC) cells, and accumulating data reveal that the optic stalk and CNC cells communicate with each other to lay the foundations for periocular and craniofacial development [10]. Development of the orbit is dependent on the interactions between the optic cup, neural crest and developing extra-ocular muscles [11]. As with other organogenesis, normal eye development relies on the spatiotemporal activation of transcription factors and the corresponding molecular signalling [7]. It is known that Pax6 must be correctly expressed for the lens and optic vesicle to develop normally, with Pax6 mutations leading to complex ocular malformations [12]. Previous studies have shown that caffeine can be transferred into the developing embryo from the external environment [13] and accumulate in the foetal brain [14,15]. Therefore, it is conceivable that an excess maternal consumption of caffeine might disrupt the normal processes of eye development as a teratogen.

Chick embryos are commonly used in developmental biology studies because of their simplicity, similarity to early stage human embryos, and ease of manipulation. In addition, early chick embryos are relatively sensitive to external physiochemical compounds such as caffeine as previously reported [16,17]. In this study, we exposed HH5 or HH10 chick embryos to different concentrations of caffeine (5, 10 and 15 μmol/egg respectively) to establish whether it could affect eye development. We investigated the effect of excess caffeine on optic development, eye morphology and expression of Pax6, Neurofilament (NF) and HNK-1 by neurons and neural crest cells during differentiation.

## Materials and methods

### Culture of chick embryos and exposure to caffeine, AAPH and Vitamin C

Fertilized white leghorn eggs were obtained from the Avian Farm of the South China Agriculture University. The eggs were incubated in a humidified incubator (Yiheng Instruments, Shanghai, China) at 38°C and 60% humidity. The embryos were allowed to develop until they reached the HH5 (only for harvest at HH9-13 stage embryos) or HH10 stage (Hamburger and Hamilton 18) and then caffeine (Nacakai, Japan) was administered by using techniques as previously described [17]. Briefly, for caffeine or AAPH exposure, different concentrations of caffeine (5, 10 and 15 μmol/egg respectively) or AAPH (10 μmol/egg) [19] were injected into the air chamber of HH5 and HH10 chick embryos. For the vitamin C rescue experiment, HH10 chick embryos were treated with 15 μmol/egg caffeine + 0.08 nmol/egg vitamin C for 7.5 days. After the injection, the shells were sealed with clear sealer tape and returned to the incubator for between 12 hrs and 7.5 days for caffeine-treated embryos and 4.5 days for AAPH-treated embryos. Control embryos were injected with the same volume of avian saline (0.72% sodium chloride). The experiments were performed in triplicate with 25 eggs assigned for each treatment group. The mortality rate was recorded during the experiment, and surviving embryos were harvested for assessment of other parameters.

### Immunohistochemistry

Whole-mount embryos and embryo sections were immunofluorescently stained with antibodies against NF, Pax6 and HNK-1 as previously described [20]. Briefly, the specimens were incubated with monoclonal antibody: Mouse anti-NF (1:200, catalogue no. 13-0700; Invitrogen, Carlsbad, CA, USA), PAX6 (1:100; DSHB, Iowa, IA, USA) or HNK-1 (1:200, catalogue no. C 0678; Sigma-Aldrich, St.Louis, MO, USA) at 4°C for 36 hrs with shaking. The specimens were treated with Alexa Fluor 488 antimouse IgG (1:1000, catalogue no. A31619; Invitrogen) or Alexa Fluor 555 antimouse IgM (1:1000, catalogue no. A21426; Invitrogen) secondary antibodies. All the specimens were counterstained with DAPI (4′-6-Diamidino-2-phenylindole, 1:1000, catalogue no. D3571; Invitrogen) for 1 hr at room temperature.

### Toluidine blue stain

Toluidine blue stain was performed on transverse sections of the embryo according to standard protocol. Briefly, paraffin sections of the embryo's head were cut at 4 μm (Leica RM2126RT). A metal loop was used to collect the sections, which were transferred to a drop of distilled water on a glass slide. The sections were then completely dried on a warm plate and a few drops of toluidine blue solution were added to the sections for 2 min. Excess dye was rinsed off the sections with distilled water. The slides were air-dried and mounted with a coverslip.

### Measurement of MDA and SOD activities

The level of oxidative stress was determined by measuring the level of malondialdehyde (MDA) and superoxide dismutase (SOD) activities in the retina of control and caffeine-treated embryos. Reactive oxygen species (ROS) production was detected with 5 μmol 2′,7′-dichlorofluorescein-diacetate (DCFH-DA, Sigma-Aldrich), which is a sensitive intracellular probe. The HH10 chick embryos were incubated for further 7.5 days after saline and caffeine (15 μmol/egg) treatment. The retinas were isolated from these embryos and homogenized for MDA and SOD assay. The MDA and SOD content were determined according to manufacturer's instructions (MDA test box, A003-1 and SOD test kit, A001-1; Nanjing Jiancheng, Nanjing, China). Protein contents were determined by using a Coomassie brilliant blue kit (Beijing Dingguo, Beijing, China). The oxidation of DCFH by ROS was established by measuring the mean fluorescence intensity of DCFH by using a Labsystems Fluoroskan Ascent plate reader (Ani Labsystems Ltd., Vantaa, Finland). The data generated from control and caffeine-treated samples were analysed according to differences in their respective fluorescein decay curves.

### Photography

Following immunofluorescent staining, the whole-mount embryos were photographed by using a stereo-fluorescence microscope (Olympus MVX10; OLYMPUS, Tokyo, Japan) and processed with a Olympus software package Image-Pro Plus 7.0. The embryos were then sectioned into 15-μm thick slices by using a cryostat microtome (Leica CM1900; LEICA, Solms, Germany) and photographed by using an epi-fluorescent microscope (Olympus IX51, Leica DM 4000B) at a magnification of 200 ×  and 400 × . The images were analysed and processed by using a CW4000 FISH Olympus software package.

### Statistical analysis

The diameter of the eye and the thickness of the retina were quantified with Image-Pro Plus 7.0 in whole-mount pictures and sections, respectively. The data were presented as mean ± standard error. Statistical analysis was performed with the spss 13.0 statistical package program for Windows (SPSS, Chicago, IL, USA). Statistical significance was determined by using one-way anova. *P* < 0.05 was considered to be statistically significant.

## Results

### Excess caffeine exposure causes development of eye malformations

**Figure 1:**
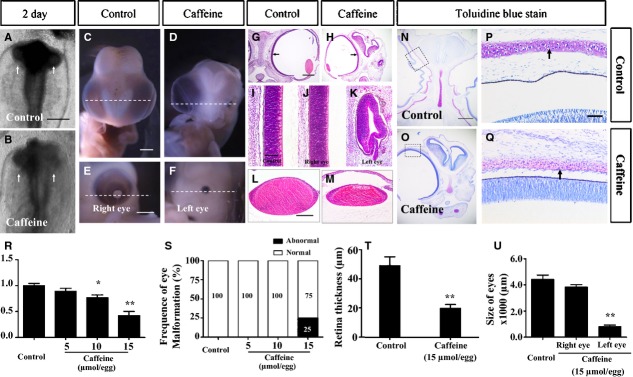
Caffeine exposure causes eye dysplasia in early chick embryos. HH5 chick embryos were injected with 15 μmol/egg caffeine for 12 hrs or HH10 chick embryos were injected with 5–15 μmol/egg for 7.5 days. All images are derived from control and 15 μmol/egg caffeine-treated embryos. (A and B) Head regions of 2-day-old control (A) and caffeine-treated (B) chick embryos. The optic vesicles are indicated by white arrows. (C and D) Ventral view of embryonic 9-day-old control (C) and caffeine-treated (D) head regions, showing excess caffeine induces unilateral microphthalmia. (E and F) Higher magnification of D showing the normal right eye (E) and smaller left eye (F). (G and M) Haematoxylin and eosin staining of transverse sections from C and D (dotted white line), showing the histology of control (G) and caffeine-treated (H) embryo head regions. (I) Histology of the control retina in G. (J and K) Appearance of the caffeine-treated retinas and lenses in the right (J and L respectively) and left (K and M respectively) of H. (N and O) Toluidine blue stained transverse sections (white dotted lines) of C and D respectively. (P and Q) Framed regions in N and O respectively, showing the orbital bone (black arrows) in the caffeine-treated embryos are less developed when compare with those of control embryos. (R–U) Bar charts showing caffeine significantly reduced the weight of the developing embryos (R), significantly increased the incidence of eye malformation (S), decreased the thickness of the whole retina (T) and reduced the size (diameter) of the eyes (U). *P ≤ 0.05 and **P ≤ 0.01 are significant difference between control and caffeine-treated embryos. Scale bars = 500 μm in A and B; 2 mm in C and D, E and F; 1 mm in G and H, N and O; 50 μm in I and K; 100 μm in L and M, P and Q.

In a previous caffeine study [17], we discovered some embryonic phenotypes with eye malformation following excess caffeine exposure. To follow up this investigation, we exposed HH5 or HH10 chick embryos to 5, 10 and 15 μmol/egg caffeine for either 12 hrs or 7.5 days. The effect of caffeine exposure was evident after 12-hour treatment in 2-day-old embryos. We observed abnormal development of chick optic vesicles which was presented as a lost of smooth outline/shape of the optic vesicle (white arrows) in the caffeine-treated embryos (Fig.1B) compared with the control (Fig.1A). The phenotype was more obvious when the embryos were examined 7.5 days after caffeine treatment (9-day-old embryo, Fig.1C–F). This was most obvious when the eye symmetry of control embryos (Fig.1C) were compared with the highest dose of caffeine (15 μmol/egg; the eye malformation occurred with a frequency of 25% approximately), which caused abnormal left and right eyes to form (Fig.1D–F, S; *N* = 10). We examined transverse sections of both control and caffeine-treated eyes (Fig.1G and H). Excess caffeine caused small eyes to develop in these embryos, the shape of the retina was altered (Fig.1I–K) and there was also a smaller lens [Fig.1L, M, U; Control = (4.42 ± 1.05) × 10^3^ μm, Caffeine Right eye = (3.83 ± 0.61) × 10^3^ μm, Caffeine Left eye = (0.79 ± 0.43) × 10^3^ μm, *N* = 10; **P < 0.01]. Furthermore, the thickness of the retina was reduced in caffeine-treated embryos (Fig.1G–K and T; Control = 49.31 ± 19.07 μm, 15 μmol/egg = 19.82 ± 8.41 μm, *N* = 20; **P < 0.01). By using toluidine blue staining, we demonstrated that the orbital bone of caffeine-treated embryos was less mature as compared with the control (as indicated by pink instead of blue staining of the orbital bone). This suggests that development of the orbital bone was impaired by caffeine exposure (Fig.1N–Q). In addition, the weight of the embryos were reduced in the caffeine-treated group (Fig.1R; Control = 1.00 ± 0.11 g, 5 μmol/egg group = 0.89 ± 0.13 g, 10 μmol/egg group = 0.76 ± 0.12 g, 15 μmol/egg group = 0.42 ± 0.17 g, *N* = 10; *P < 0.05, **P < 0.01). Taken together, our data suggest that excess caffeine exposure leads to morphological malformation of embryonic eyes.

### HNK-1 and NF expression in caffeine-induced eye malformations

**Figure 2:**
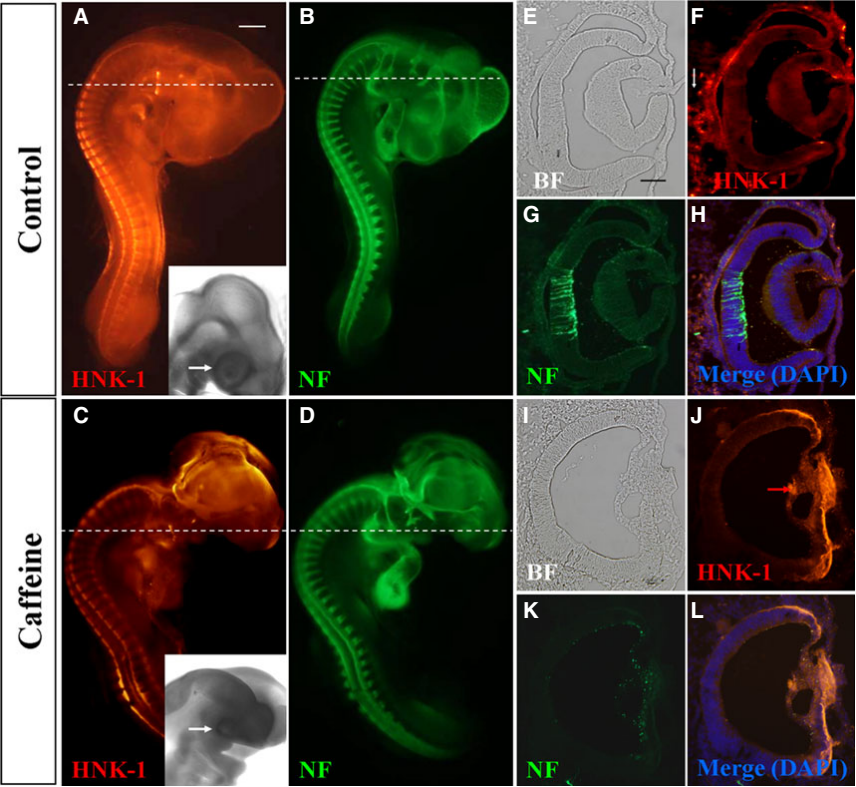
Caffeine exposure impaired neurofilament (NF) and HNK-1 expression in early chick eyes. HH10 chick embryos were treated with 15 μmol/egg caffeine for 1.5 days and then whole-mount double immunostained with NF and HNK-1 antibodies. (A–D) Control and caffeine-treated embryos stained with HNK-1 (A and C) and NF (B and D) antibodies. Higher magnified bright-field images indicated the eye malformation induced by caffeine (black arrows). (E–H) Transverse sections of A and B (dotted white lines) showing the expression pattern of HNK-1 and NF in the control. There are some HNK-1^+^ neural crest cells surrounding the eyes in control embryo (white arrow in F), but absent in caffeine-treated ones. (I–L) Transverse sections of C and D (dotted white lines) showing HNK-1 and NF expression in caffeine-treated eyes is distinctly different from control eyes. BF, bright-field. Scale bars = 1 mm in A–D; 100 μm in E–L.

The retina is derived from the optic vesicle and later the optic cup, which themselves are originally derived from the embryonic neural plate. HNK-1 is mainly expressed in migratory neural crest cells which contribute to the head mesenchyme [7,21]. Therefore, we performed double immunofluorescent staining to determine NF and HNK-1 expression in control and caffeine-treated embryonic eyes (Fig.2A–D). We can find the eye malformation induced by caffeine in higher power bright-field images (white arrows in insets of Fig.2A and C). In cross sections produced from these eyes, we found distinct morphological differences between control (Fig.2E) and treated embryos where the lens was missing (Fig.2I). We found ectopic HNK-1^+^ neural crest cells in the anterior segment of the caffeine-treated eye compared with the control embryos (Fig.2F, H, J and L). Neurofilament expression in the retina was also examined and found to be affected by excess caffeine exposure. The NF^+^ cells were presented as a scattered pattern in the retina (Fig.2K and L) compared with highly ordered NF^+^ cells in the control (Fig.2G and H). These findings suggest that excess caffeine exposure affected neural crest cell migration around the embryonic optic cup and interfered with NF^+^ retinal neurogenesis.

### Excess caffeine altered Pax6 expression in the developing eye

**Figure 3:**
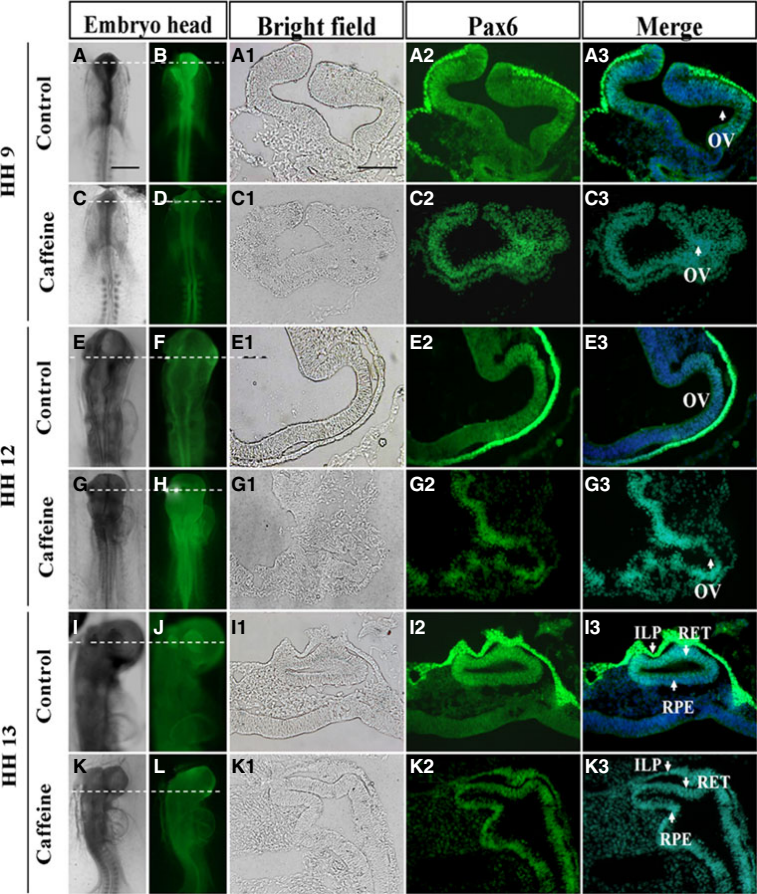
Pax6 expression pattern in the chick optic vesicle and neuroepithelium is perturbed by caffeine. Whole-mount immunostaining was performed in control and caffeine-treated HH9 (A–D), HH12 (E–H) and HH13 (I–L) chick embryos respectively. (A1–K3) Transverse sections of A–L (indicated by dotted white line) showing the spatiotemporal expression pattern for Pax6 in the developing eye. OV: optic vesicle; ILP: invaginating lens placode; RET: retina; RPE: retinal pigment epithelium. Scale bars = 500 μm in A–L, and 100 μm in A1–K3.

Pax6 acts as a master transcriptional gene that regulates the formation of the optic vesicle/cup and lens placode [7,22]. In this section of the study, we firstly detected the spatiotemporal expression pattern for Pax6 during eye development in HH9-13 chick embryos (Fig.3A, B, E, F, I and J). In whole-mount embryos, Pax6 was found to be mainly expressed in the optic vesicle and lens (Fig.3B, F, J). Transverse sections of these eyes revealed that Pax6 was strongly expressed in the neuroepithelium and optic vesicle of early embryos (Fig.3A2-A3, E2-E3) and also in the retinal anlagen of older embryos (Fig.3I2-I3). However, the expression of Pax6 in optic vesicle and neuroepithelium was more broadly expressed after caffeine exposure of early embryos (Fig.3C2-C3, G2-G3) and also in the retinal anlagen of older embryos (Fig.3K2-K3).

In the HH9 control embryo (Fig.3A2-A3), Pax6 protein is most abundant in the surface ectoderm (presumptive lens ectoderm), and is more weakly expressed in the distal portion of the optic vesicle contacting the surface ectoderm. In the HH9 caffeine-treated embryo (Fig.3C2-C3), Pax6 protein cannot be detected in the surface ectoderm, but its expression in the neuroepithelium of the optic vesicle is more widespread and is not restricted to the distal portions only (Fig.3C3, white arrow).

In the HH12 control embryo (Fig.3E2-E3), Pax6 protein is also abundantly expressed in the surface ectoderm, while in the optic vesicle it is restricted to a dorsal/distal domain. In the corresponding caffeine-treated embryo (Fig.3G2-G3), the optic vesicle is highly disorganized providing the first indication of aberrant morphogenesis. Pax6 protein is distributed ectopically within the treated optic vesicle (Fig.3G3, white arrow). In the surface ectoderm of this embryo, Pax6 protein expression is now visible, but is highly restricted to a small region of ectoderm contacting the underlying optic vesicle. It is possible that this delayed expression may have been induced by the optic vesicle but may not be sufficient to initiate normal lens development.

In the HH13 control embryo (Fig.3I2-I3), Pax6 protein is still abundantly expressed in the surface ectoderm as well as in the retinal anlagen. In the corresponding caffeine-treated embryo (Fig.3K2-K3), the retinal anlagen is not well developed and Pax6 protein expression is still disturbed (Fig.3K3, white arrow). On the surface ectoderm of this embryo, Pax6 protein expression level in the retinal anlagen is not much higher than the one in the control embryo (Fig.3K3, white arrow).

**Figure 4:**
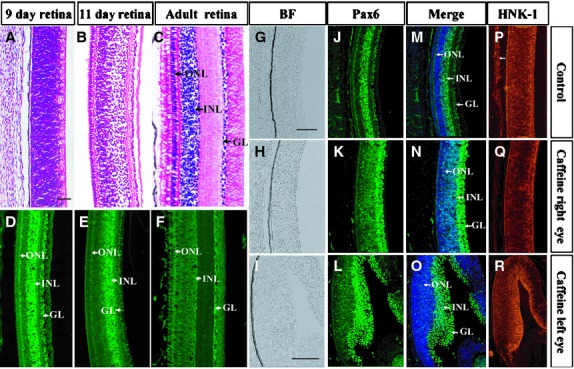
Pax6 expression pattern in the chick retina is perturbed by caffeine. Haematoxylin and eosin staining and Pax6 & HNK-1 immunostaining were performed on various developmental stage of chick retina. (A–F) Transverse sections of normal embryonic 9-day (A and D), 11-day (B and E) chick embryo and adult chick (C and F) retina stained with haematoxylin and eosin and Pax6 respectively. (G–R) Embryonic 9-day-old control (G–P) and caffeine-treated (H–R) retina stained with Pax6 and HNK-1 antibodies. Sections were counterstained with DAPI. The distribution of Pax6^+^ cells in caffeine-treated retina (N and O) is distinctly different from that of normal retina (M). INL, inner nuclear layer; ONL, outer nuclear layer and GL, ganglion cell layer. Scale bars = 500 μm in A–F, 100 μm in G and H, J and K, M and N, P and Q and 100 μm in I, L, O, R.

Interestingly, it appeared from the staining pattern that that Pax6 might be involved in the differentiation of retinal layers when the embryo reached embryonic 9-day. Pax6 expression was distinctly distributed in the three main cell layers of the embryonic 9-day-old retina (Fig.4D), although there were no discernible morphologically boundaries as revealed by haematoxylin and eosin staining of the retina (Fig.4A). We observed that Pax6 expression was gradually restricted to the inner nuclear layer (Fig.4E) at 11-day when the different layers in the retina are clearly distinguishable (Fig.4B). To obtain a more obvious interpretation of our findings, we decided to also establish Pax6 expression in the retina of adult chick eyes (Fig.4F). In these eyes, haematoxylin and eosin staining revealed that the inner and outer nuclear layers, as well as the ganglion cell layer were clearly distinguishable (Fig.4C). We compared the Pax6 expression pattern between caffeine-treated and control retina in the 9-day-old embryos (Fig.4G, J, M), and established that Pax6^+^ cells were distributed as a band of cells directly opposing at the lumen of the retina in the right eye (Fig.4H, K, N) and in the left eye of caffeine-treated eyes (Fig.4I–O).

### Excess ROS production is involved in caffeine-induced eye dysplasia

**Figure 5:**
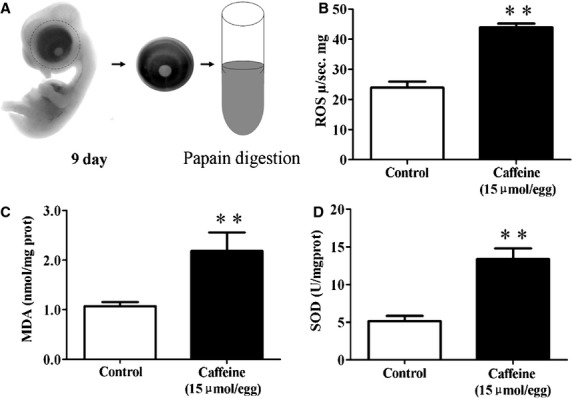
Caffeine exposure increased oxidative stress in the developing eyes. Reactive oxygen species (ROS) production and MDA & SOD activities were determined in the 9-day chick embryonic eyes following caffeine exposure. (A) Schematic chart showing the acquisition of eye tissue from 9-day-old chick embryos for analysis. (B) Bar charts showing caffeine (15 μmol/egg) significantly increased ROS production as compared with the control. (C and D) Caffeine (15 μmol/egg) also significantly increased MDA & SOD activities (**P < 0.01).

It has been reported that caffeine could directly elevate intracellular oxidation stress [23,24]. Therefore, we investigated the level of ROS production and anti-oxidation ability in the context of the caffeine-treated eye, as shown schematically in Figure5A. Our results showed that ROS generation was significantly increased in 9-day chick embryo eye tissues after the 15 μmol/egg caffeine treatment (Fig.5B; Control = 23.90 ± 5.78 μ/s mg, caffeine-treated group = 43.94 ± 3.50 μ/s mg, *N* = 8; *P* < 0.01). Assay for MDA revealed that there was an increase for the caffeine-treated groups (Fig.5C; Control = 1.07 ± 0.16 nmol/mgprot, caffeine-treated group = 2.18 ± 0.70 nmol/mgprot, *N* = 8; *P* < 0.01). Similar result was observed for SOD activity, which is an anti-oxidation indicator (Fig.5D; Control = 5.16 ± 1.26 U/mgprot, caffeine = 13.78 ± 2.64 U/mgprot, *N* = 8; *P* < 0.01). All these data indicate that ROS accumulation correlates with caffeine-induced eye dysplasia.

**Figure 6:**
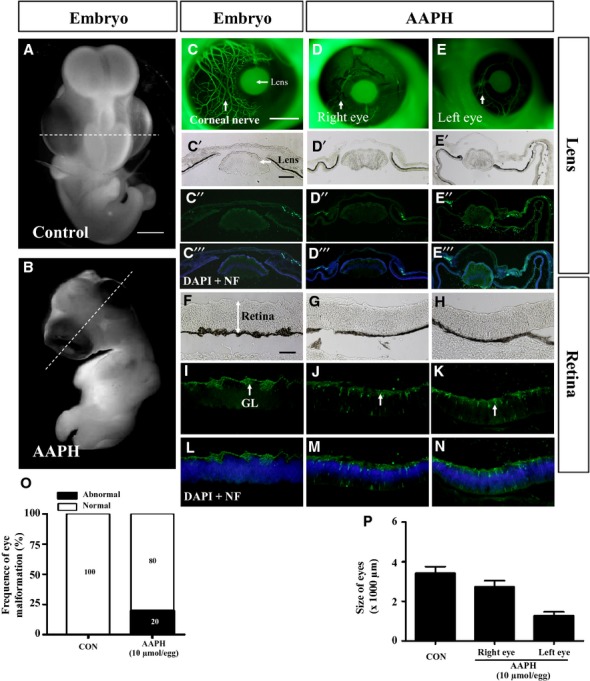
AAPH exposure replicates the effect of caffeine on the developing eye. Early chick embryos exposed to 10 μmol/egg AAPH for 6 days and immunostained with NF antibody. (A and B) Control (A) and AAPH-treated (B) chick embryos. (C–E) NF immunostaining demonstrates the presence of neurons in control (C) and AAPH-treated (right eye, D) and (left eye, E) lens. The staining shows AAPH-treatment repressed neuron development in the eyes. (C'–E''') Transverse section of the lens at the levels indicated by white line in A and B. Bright-field (C', D', E'), NF immunostaining (C'', D'', E'') and NF immunostaining + DAPI (C''', D''', E'''). (O and P) Bar charts showing AAPH significantly increased the incidence of eye malformation (O) and reduced the size (diameter) of the eyes (P). NF, neurofilament. Scale bars = 2 mm in A and B; 1 mm in C–E; 200 μm in F–N.

To establish causality between ROS elevation and eye dysplasia, we used 2,2′-azobis (2-amidinopropane) dihydrochloride (AAPH) which has been extensively used as a free radical generator [19]. In the excess AAPH-treated chick embryos, we obtained a similarly abnormal phenotype as in caffeine-treated embryos—asymmetrical eyes developed in 20% of embryos (Fig.6B, D, E and O) compared with the control (Fig.6A, C and O). The average diameter of the eyes in the control group is distinctly larger than that of the AAPH-treated group [Fig.6D, E and P; Control = (3.32 ± 0.95) × 10^3^ μm, AAPH Right eye = (2.86 ± 0.83) × 10^3^ μm, AAPH Left eye = (1.29 ± 0.52) × 10^3^ μm, *N* = 10; **P < 0.01). It is worth nothing that neurogenesis occured between the epithelium and lens in the developing eye was repressed in following AAPH treatment (Fig.6D–D''' and E–E''') as compared with the control (Fig.6C–C'''). Neurofilament immunostaining positively identified the neurons in the corona (Fig.6C–E). Furthermore, the corneas of AAPH-treated eyes (Fig.6D and E) were not as transparent as in the control (Fig.6C). The NF-labelled neurons were restricted to ganglion layer in the control retina (Fig.6G and H) but were not so restricted after AAPH treatment (Fig.6J–M, K–N).

**Figure 7:**
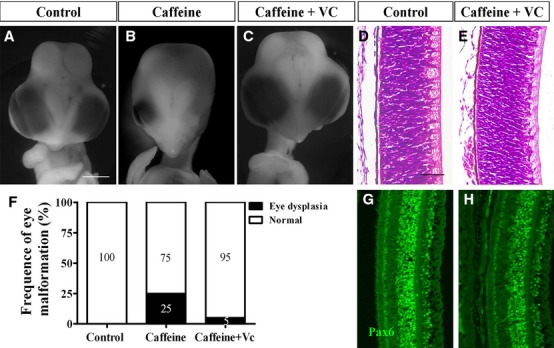
Addition of vitamin C rescues caffeine-induced eye malformation. HH10 chick embryos were treated with 15 μmol/egg caffeine or 15 μmol/egg caffeine + 0.08 nmol/egg vitamin C for 7.5 days and immunostained with Pax6 antibody. (A–C) Representative appearance of control (A), caffeine (B) and caffeine + vitamin C-treated (C) embryos. The caffeine + vitamin C-treated embryos were morphologically indistinguishable from control embryos. (D and E) Transverse sections of control (D) and caffeine + vitamin C-treated (E) retina stained with haematoxylin and eosin. The histology shows that the retina between both groups are relatively similar in appearance. (F) Bar chart showing the incidence of eye malformation induced by caffeine is significantly reduced with the addition of vitamin C. (G and H) Control (G) and caffeine + vitamin C (H) retina immunofluorescently stained for Pax6 showing the expression between both groups are relatively similar. Scale bars = 2 mm in A–C; 50 μm in D and E and G and H.

To further validate the relationship between caffeine and ROS generation in abnormal eye development, we examined the combined effect of vitamin C and caffeine on the chick embryos. Vitamin C is a well-known antioxidant (Fig.7). We observed that the dysplastic eye phenotype was rescued by vitamin C (Fig.7C and F; 5% eye malformation, *N* = 10), whereas eye dysplasia was still evident in the caffeine-treated group (Fig.7B; 25% eye malformation, *N* = 10). Pax6 expression (Fig.7G and H) and retinal morphology (Fig.7D and E) were not different between control and caffeine plus vitamin C groups. Hence, we can reasonably speculate that caffeine exposure induces excess ROS production which in turns impairs the normal development of the eyes during early embryogenesis.

## Discussion

The general public is constantly informed of the harmful effects of alcohol and tobacco but not caffeine, despite it being consumed daily in our food and beverages. This is because caffeine consumption is perceived as being safe. However, there is now growing concern of the consequence of caffeine toxicity on foetal development because caffeine consumption has increased worldwide. We are now gradually beginning to understand the adverse effects of caffeine on foetal development [17] but a great deal of research is still needed. In this study, we investigated the potential risks of caffeine on foetal eye development, by using the chick model and immunocytochemistry, to investigate the neurons and neural crest cells associated with eye development. It has been reported that exposure to 100–600 μM caffeine affected neurogenesis in the mouse embryo [25,26]. We decided to use 5, 10 or 15 μmol/egg of caffeine (each egg is about 50 ml albumen and yolk), which represents consuming several cups of coffee, in our study and found that it reduced the weight of developing chick embryos and also induced eye malformation. Specifically, excess caffeine not only affected the formation of optic vesicle and the development of optic vesicle into retina, but also the development of lens. We also found that the eyes developed asymmetrically, in which the thickness of the retina and size of the lens were distinctly affected. In addition, the orbital bone, which is the accessory structure of the eye, was deficient after caffeine treatment. It has been reported that excess caffeine causes changes to the cornea [27,28] and the failure of neural tube closure because of excessive neuroepithelial cell proliferation [26]. In rat embryos, up to 91% of the caudal neural tubes failed to close following caffeine treatment [29]. We have previously reported that caffeine exposure could disrupt neural tube development and induce other teratogenic effects in the chick embryos [17,30]. The eyes are derived from the neuroectoderm of cranial neural tube that differentiates into the retina and associated pigment cells. The cranial mesoderm contributes to the corneoscleral and uveal tunics. In this study, we used NF as a marker to follow the development of the neural tube-derived retina and HNK-1 to track the CNC cells. We found that excess caffeine inhibited the development of the NF^+^ retina and led to the ectopic migration of HNK-1^+^ neural crest cells between the optic vesicle and presumptive lens. Bailey *et al*. previously reported that loss of NCCs leads to precocious/ectopic lens differentiation [9], so we proposed that the ectopic HNK-1^+^ neural crest cells in the anterior segment of the caffeine-treated eye (Fig.2J) was very likely to contribute to the failure of lens development *via* their well-characterized inhibitory signalling. Similarly, their ectopic migration might result indirectly from the failed optic vesicle morphogenesis as demonstrated in Figure3.

Pax6 is regarded as the true master control gene that specifies eye development in both vertebrates and insects [31–33]. When Pax6 was mutated in human it led to microphthalmia [34,35], aniridia and pan-ocular disorder [36]. We demonstrated the Pax6 protein expression pattern in the early stage of chick eye, –showing it was expressed in the optic vesicle, invaginating lens placode and the retinal pigment epithelium. Interestingly, we found that Pax6 expression in the retina was gradually attenuated from embryonic 9-day to adult eye which indicates that it might play an important role in the differentiation of the inner and outer nuclear layers, and the ganglion cell layer. Pax6 expression was disturbed in the caffeine-treated optic vesicle and presumptive lens ectoderm compared with the control in HH9, HH12, HH13 and 9-day-old embryos. We speculate that this is the principal mechanism that caused the caffeine-induced retinal dysplasia. It has already been reported that Pax6 is crucial for craniofacial skeletal development by regulating CNC cell migration [37]. In chick embryos, neural crest cells regulate the Pax6 activity of adjacent non-lens ectoderm *via* smad3 and canonical Wnt signalling [8]. Hence, the data presented in Figures2 and 3 suggested that Caffeine exposure down-regulates Pax6 protein levels in the presumptive lens ectoderm, while giving a broader distribution of Pax6 protein in the developing optic vesicle (Fig.3C2 and C3). Optic vesicle morphogenesis is greatly disturbed, possibly as a consequence of the effect on Pax6 protein distribution. This greatly compromises the association of the optic vesicle with the overlying presumptive lens ectoderm (Fig.3G2 and G3). Because of their poor association, migrating neural crest cells are able to infiltrate the region between optic vesicle and presumptive lens (Fig.2J and L). While the abnormal optic vesicle may be able to induce delayed Pax6 expression in the presumptive lens (Fig.3G2 and G3), the presence of ectopic neural crest cells (Fig.2J and L) compromises normal patterning of both lens and retina. The effects of ROS were determined at a developmental end-point, but it is possible that ROS might mediate the early (HH9-12) perturbation of Pax6 protein distribution and optic vesicle morphogenesis, since co-culture with both caffeine and Vitamin C rescued the end-point phenotype.

In our previous study, we have shown that excess ROS production by AAPH (a free redial generator) caused dysplasia of the cardiovascular system during embryogenesis [19]. Similarly, we have demonstrated that caffeine treatment also led to excess ROS generation in the developing chick eyes which induced abnormal eye dysplasia. We could mimic this abnormal phenotype by exposing the eyes to AAPH. In addition, neurogenesis between the corneal epithelium and lens was repressed following AAPH and caffeine treatments. We showed that it was possible to rescue the embryo from developing eye dysplasia, induced by caffeine treatment, by adding antioxidant vitamin C. It has been reported that the Pax6 over-expressing cells possessed a higher level of ROS [38]. Furthermore, it has also been demonstrated that in alcohol-induced foetal microcephaly, the abnormality was associated with suppression of Pax6 by ROS, suggesting an interrelation between Pax6 and ROS *in vivo* [39]. In our experiment, we have shown that abnormal Pax6 expression induced by caffeine treatment was rescued by the addition of vitamin C in the 9-day embryos. This implies that excess ROS production induced by caffeine is the key factor for disrupting Pax6 expression in the eye during development.

**Figure 8:**
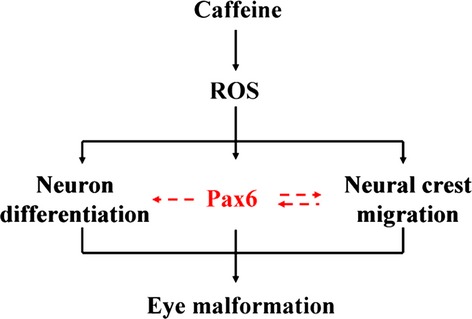
Propose model summarizing the overall effect of caffeine exposure on the developing embryonic eye. The neural crest migration and neuron differentiation was disturbed after excess caffeine exposure. These might be because of perturbed Pax6 expression. Excess reactive oxygen species (ROS) production is also involved in caffeine-induced eye dysplasia. The eye malformation and abnormal Pax6 expression induced by caffeine treatment was rescued by the addition of antioxidant. This implies that excess ROS production induced by caffeine is the key factor for disrupting Pax6 expression in the eye during development.

In sum, we propose that excess caffeine perturbs Pax6 expression, the master gene that controls eye development, by over-production of ROS in the developing eyes. The perturbed Pax6 expression in turn disrupts neuron differentiation in the retina and orbital bone (which is partly derived from CNC cells) formation, culminating in eye dysplasia. This is schematically summarized in Figure8. In this context, our findings will have important implications for pregnant mothers as over consumption of caffeine could potentially be teratogenic.
